# Embryonic Development of *Pediculus humanus capitis*: Morphological Update and Proposal of New External Markers for the Differentiation Between Early, Medium, and Late Eggs

**DOI:** 10.1007/s11686-023-00667-0

**Published:** 2023-03-15

**Authors:** Blanca E. Álvarez-Fernández, M. Adela Valero, Benjamín Nogueda-Torres, M. María Morales-Suárez-Varela

**Affiliations:** 1 Departamento de Parasitología, Facultad de Farmacia, Universidad de Valencia, Av. Vicente Andrés Estellés S/N, Burjassot, 46100 Valencia, Spain; 2 Facultad de Ciencias Químico Biológicas, Universidad Autónoma de Guerrero, Av. Lázaro Cárdenas. Ciudad Universitaria, 39090 Chilpancingo, Guerrero México; 3 Centro de Investigación Biomédica en Red de Enfermedades Infecciosas (CIBERINFEC), Instituto de Salud Carlos III, 28029 Madrid, Spain; 4 Departamento de Parasitología, Escuela Nacional de Ciencias Biológicas, Instituto Politécnico Nacional, Prolongación de Carpio y Plan de Ayala, Miguel Hidalgo, Santo Tomás, 11340 Mexico City, México; 5 Departament of Preventive Medicine and Public Health, Sciences of Eating, Toxicology and Legal Medicine, Faculty of Pharmacy, Universidad de Valencia, Av. Vicente Andrés Estellés, Burjassot, 46100 Valencia, Spain; 6 Consorcio para la Investigación Biomédica en Red de Epidemiología y Salud Pública (CIBERESP), 28029 Madrid, Spain

**Keywords:** *Pediculus humanus capitis*, Defective eggs, Embryonic development, Yolk granules

## Abstract

**Background and Objectives:**

The head louse *Pediculus humanus capitis* is a cosmopolitan ectoparasite that causes pediculosis. In the study of human lice, little research focuses on embryonic development. Currently, external markers of embryonic development represent a new approach in the evaluation of ovicidal drugs. The objective of this work was to update the morphology of embryonic development and propose novel external markers to differentiate between early, medium, or late *P. h. capitis* eggs.

**Methods:**

Using stereoscopic light microscopy, we describe the morphological characteristics of *P. h. capitis* eggs with a special focus on embryonic development.

**Results:**

The morphological analysis of the eggs revealed the presence of an operculum with ten aeropyles, although no micropyles were observed. For the first time, the presence of defective eggs that were non-viable due to the apparent absence of yolk granules was documented. The early eggs presented yolk granules and developing germ bands, while the medium eggs presented an embryonic rudiment and the outlines of the eyes and limbs. In late eggs, the head with eyes and antennae, the thorax with three pairs of legs, and the abdomen with six pairs of spiracles were observed as formed structures. At the end of this stage, the embryos acquired the morphology of the nymph I stage.

**Conclusion:**

We propose novel biomarkers (e.g., the presence of spiracles and antennae, the proportion of the egg occupied by the embryo) to facilitate the differentiation between the developmental stages. The updated morphological characteristics of *P. h. capitis* eggs facilitate the standardization of toxicological tests in the quest for ovicidal drugs.

## Introduction

The head louse *Pediculus humanus capitis* (Phthiraptera: *Pediculidae*) (De Geer, 1767) is a cosmopolitan ectoparasite that causes pediculosis capitis [1]. *P. h.* capitis which is a hematophagous (i.e., feeds on blood) hemimetabolous insect, lives and is reproduced on the human head [2], where it accesses nutrients directly from the venules of the scalp [3].

The diagnosis of head lice infestation is based on the finding of living lice in the hair of a person during any one of their developmental stages, i.e., egg, nymph, or adult [4], with the egg being the most frequently detected form. The use of a fine-toothed detection comb improves the efficiency of lice sampling [5–7]. Head lice infestation is relatively easy to treat with the use of topically applied agents that eliminate the insects and their eggs.

The adult female lives for about a month, when it produces approximately 300 eggs [8] that are oviposited at the base of hair shafts [2]. The eggs’ average size is 0.8 mm by 0.3 mm; they have an ovoid shape and generally present a shade of yellow or white [9]. A vault-like operculum is found on the distal end of the egg [10]. The operculum has 7–11 aeropyles that occupy half of its surface [10]. During oviposition, females secrete a liquid that is then solidified, forming the nit sheath [11]. The nit sheath proteins LNSP1 and LNSP2 are essential for maintaining egg viability and also function as a glue [12]. The only part of the egg free of glue is the operculum, which protects the embryo from asphyxiation during development [11, 12].

In insects, the female must provide the eggs with sufficient nutrients to sustain embryogenesis [15]. To this end, the fatty body synthesizes massive amounts of proteins that are secreted into the hemolymph to reach the ovaries, where they are incorporated into the developing oocytes [15]. Once inside the oocyte, proteins accumulate in granules, called yolk granules [15]. This process is called vitellogenesis and normally occurs only in the terminal oocyte; subsequently, the yolk membrane, chorion, and aeropyles are formed [16].

Embryogenesis varies among insect groups or even among closely related species. In hemimetabolous species, it usually starts with the formation of the blastoderm and the germ band and terminates with organogenesis [17]. During the embryonic development of insects, events related to cellular differentiation, growth, and morphogenesis occur [18]. Since these events are crucial, toxicokinetic and toxicodynamic processes are modified during embryogenesis. These toxicological variations have been reported in several species [17–21]. In the study of human lice, little research has focused on embryonic development [22–24]. However, markers of embryonic development currently represent a new approach to assess the toxicity of insecticides against human lice eggs [25].

Studies on the activity of pediculicidal products against eggs use different criteria to evaluate the effect on their development. In this context, viability criteria have been used to define eggs into the following categories: (a) alive, i.e., the egg is located at approximately < 1 cm from the scalp, the operculum is closed, the egg has a uniform ovoid shape and density, and an “eye spot” may appear depending on the age of the egg, (b) dead, i.e., the egg has a misshapen, shriveled, indented, or irregular shape, or the egg has a non-uniform density with parts of the egg being clear, whereas other parts of the egg are opaque, or (c) hatched, i.e., the operculum is open and the nymph is not in the egg [27]. Other studies on the efficacy of pediculicides are based on embryological development criteria such as changes in the appearance of *P. h. capitis* embryos assessed at different stages [25]. Based on the color of the ocular spots and the appearance of appendages, the developing eggs of head and body lice were previously divided into three stages: early, medium, and late. Early eggs are characterized by the absence of external markers, while medium eggs present reddish eyes and outlines of the appendages, and late eggs have developed black eyes and visible appendages [25].

The objective of this work was to update the morphology of embryonic development and propose novel external markers to differentiate between early, medium, or late *P. h. capitis* eggs that were obtained by natural infestation. To this end, stereoscopic light-field microscopy was applied. The updated morphological characteristics of the egg will facilitate the standardization of toxicological assays in the quest for ovicidal drugs.

## Materials and Methods

### Ethical Statement

For the collection of specimens, the necessary permits were obtained from the Municipal and School Authorities. Informed consent was obtained from the participants’ parents before the study was initiated. Verbal consent from the participants was also required. The collection was carried out in accordance with the Declaration of Helsinki.

### Participants and Sample Collection

To identify the characteristics of the three embryonic developmental stages, *P. h. capitis* eggs (*N* = 722) were obtained from 50 girls aged 7–14 years. The collection took place from June to December 2019 in the city of Chilpancingo (Guerrero, Mexico). Eggs detached from and attached to the hair at distances of 0.5–6.0 cm from the scalp were collected by dry combing with a fine-toothed metal comb. Participants who suffered a chronic dermatological disease or had used pediculicides or antibiotics in the past 6 months were excluded.

The samples from each participant were placed in individual Eppendorf tubes filled with Karnovsky fixative (25% paraformaldehyde; 0.5% glutaraldehyde) at the moment of collection. The samples were kept at room temperature (between 17° and 20° C). The tubes were sealed with Parafilm and placed on a test-tube rack with hermetically sealed cover for transportation. A stereoscopic microscope (Nikon SMZ-U; Nikon Instruments, Tokyo, Japan) was used for the morphological analysis. Morphological identification was carried out from January to March 2020 in the Parasitology Laboratory of the Department of Parasitology of the University of Valencia (Spain). For this study, 651 eggs detached from the hair and 71 eggs attached to the hair (*N* = 722) were analyzed.

### Stereoscopic Microscopy

The 722 eggs were individually mounted in Petri dishes with Karnovsky fixative to avoid dehydration. To visualize the specimens, the ×0.75 and ×7.5 objectives were used, with the focus and light individually adjusted to observe the external morphology of the egg and through the chorion to search for embryonic development characteristics. The eggs were classified into three developmental stages according to previously reported characteristics of human lice [22–27] and the cricket *Gryllus bimaculatus*, a hemimetabolous insect used as a study model in neurobiology, physiology, and genetics [30]. All eggs were photographed and subsequently stored in Eppendorf tubes with fresh Karnovsky fixative.

## Results

Of the total number of eggs (*N* = 722), 550 (76.17%) were considered viable based on a complete external morphology and the presence, to a greater or lesser extent, of some embryogenesis characteristics. Of the remaining 172 eggs, 168 (23.26%) had hatched and 4 (0.55%) had an intact external morphology. However, they did not contain yolk. Thus, these eggs were non-viable by default. Hereinafter, these are called defective/non-viable eggs.

The viable eggs (*N* = 550) were grouped into three developmental stages according to their individual characteristics: early (*N* = 138; 19.11%), medium (*N* = 208; 28.80%), and late (*N* = 204; 28.25%) eggs. The eggs presented a semi-transparent, amber coloration that permitted the observation of the external morphology and characteristics.

### Egg Morphology

**Fig. 1:**
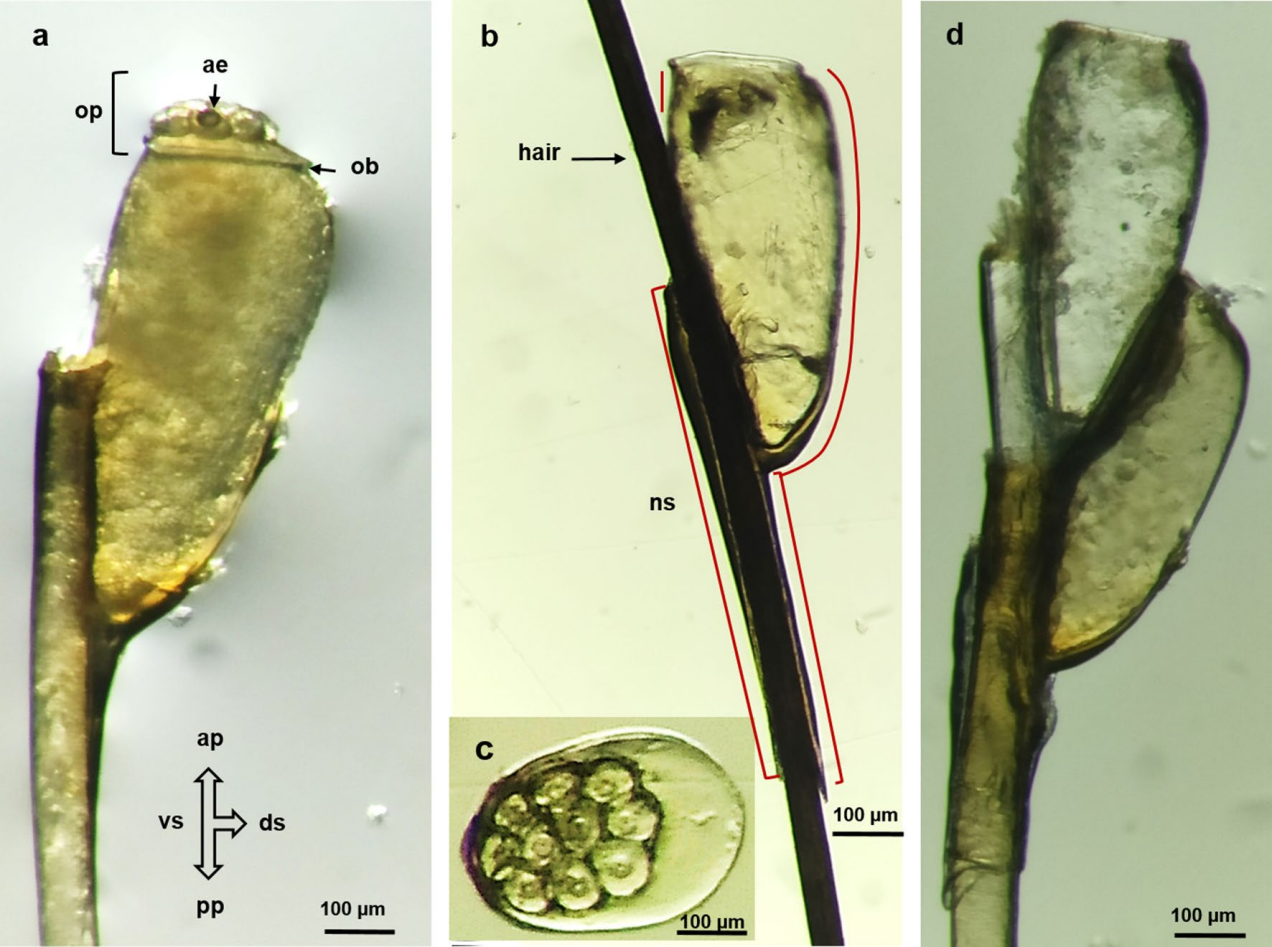
Morphology the *P. h. capitis* egg under stereoscopic microscopy. **a** Fertile egg with visible ventral (vs) and dorsal sides (ds), the anterior (ap) and posterior poles (pp), the opercular pole (op), the opercular border (ob), the operculum (op), and the aeropyles (ae); 100× magnification. **b** Hatched egg. The nit sheath (ns) can be observed, most notably in the apical part of the egg, beside the hair (orange lines). 100× magnification. **c** Detail of the operculum with ten aeropyles; 100× magnification. **d** Hair with two hatched eggs; 100× magnification

The eggs of *P. h. capitis* had an ovoid shape with an operculated anterior pole and a posterior pole presenting a blunt termination. The ventral side was semi-flattened, and the dorsal side was concave. The last third of the eggs was ventrally attached to the hair, forming nit sheaths of variable length that partially surrounded the hair (Fig. 1a). In some cases, the glue was more abundant on the apical part of the egg. The operculum, with a defined opercular edge, was convex and presented ten round protuberant cells with a central orifice, known as aeropyles. The aeropyles were arranged concentrically on the ventral side of the egg (Fig. 1b–c). The surface of the chorion was smooth and undecorated. Except for the operculum, no other orifices corresponding to micropyles were observed.

In most of the participants with mild or moderate infestations, one egg per hair was found. However, in the case of severe infestation, two viable or hatched eggs per hair were found (Fig. 1d).

### Defective/Non-viable Eggs

**Fig. 2:**
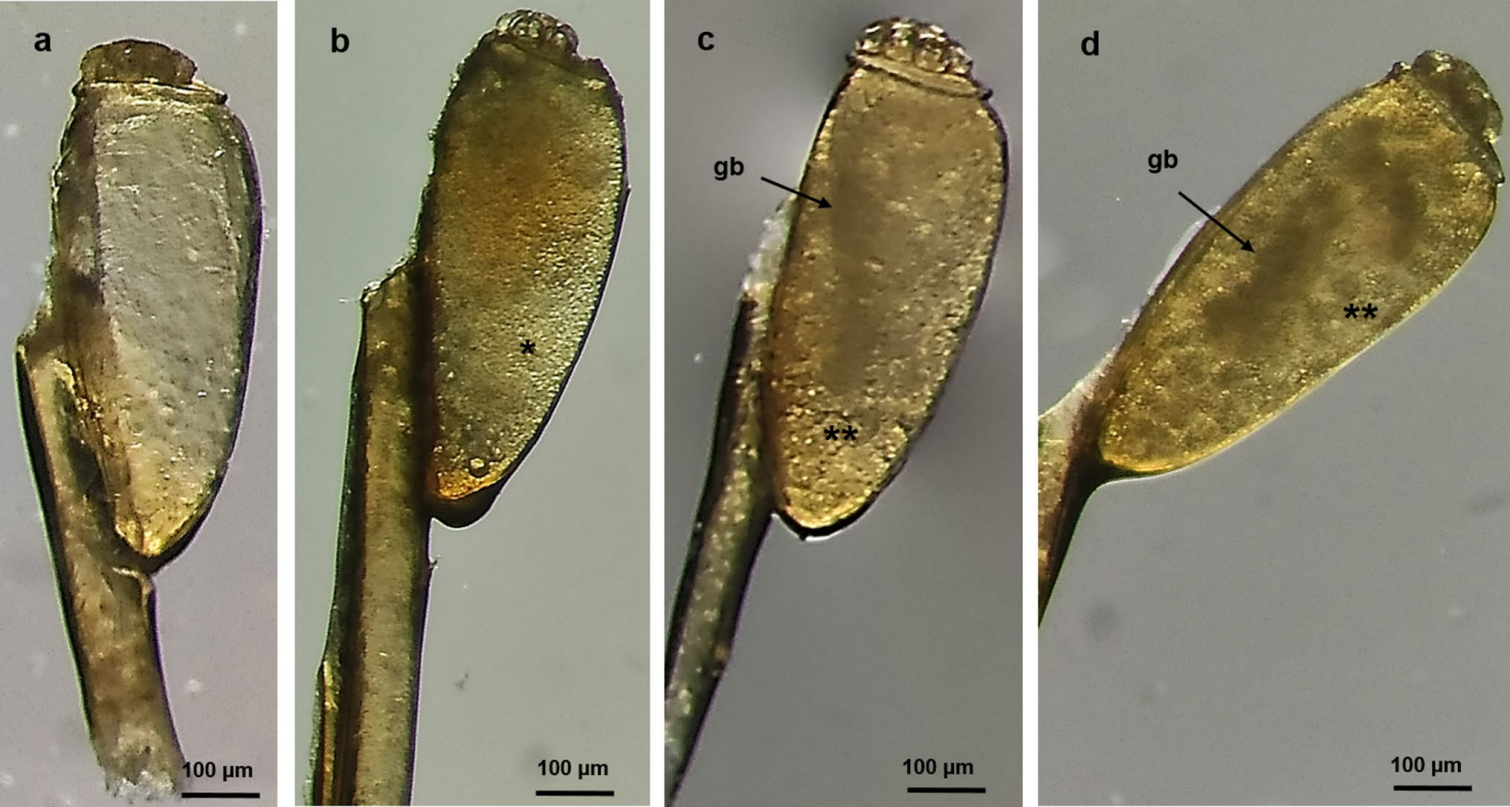
Morphology of defective/non-viable and early *P. h. capitis* eggs. **a** The defective/non-viable egg does not present yolk; 100× magnification. **b–d** Early eggs. **b** Egg with abundant fine yolk granules (*); 100× magnification. **c** Egg with multiple yolk granules of variable size (**) and a forming germ band (gb); 100× magnification **d** Egg with yolk granules relocated to the center (**) and an evident germ band (gb); 100× magnification

These eggs presented the external morphology of a viable egg, without differences on a macroscopic level. Nevertheless, no internal structures were visible: the eggs appeared empty and without yolk. The defective/non-viable eggs presented a sealed operculum (Fig. 2a), unlike the hatched eggs (Fig. 1d).

### Morphology of the Embryogenesis

#### Early Eggs

At this developmental stage, the eggs presented qualities corresponding to early embryogenesis. Eggs with abundant fine yolk granules (Fig. 2b), eggs with multiple yolk granules of variable size with a forming germ band (Fig. 2c), and eggs with yolk granules relocated to the center and an evident germ band (Fig. 2b–d) were analyzed. The presence of fine yolk granules allowed the differentiation between defective and early eggs (Fig. 2a–b).

#### Medium Eggs

**Fig. 3:**
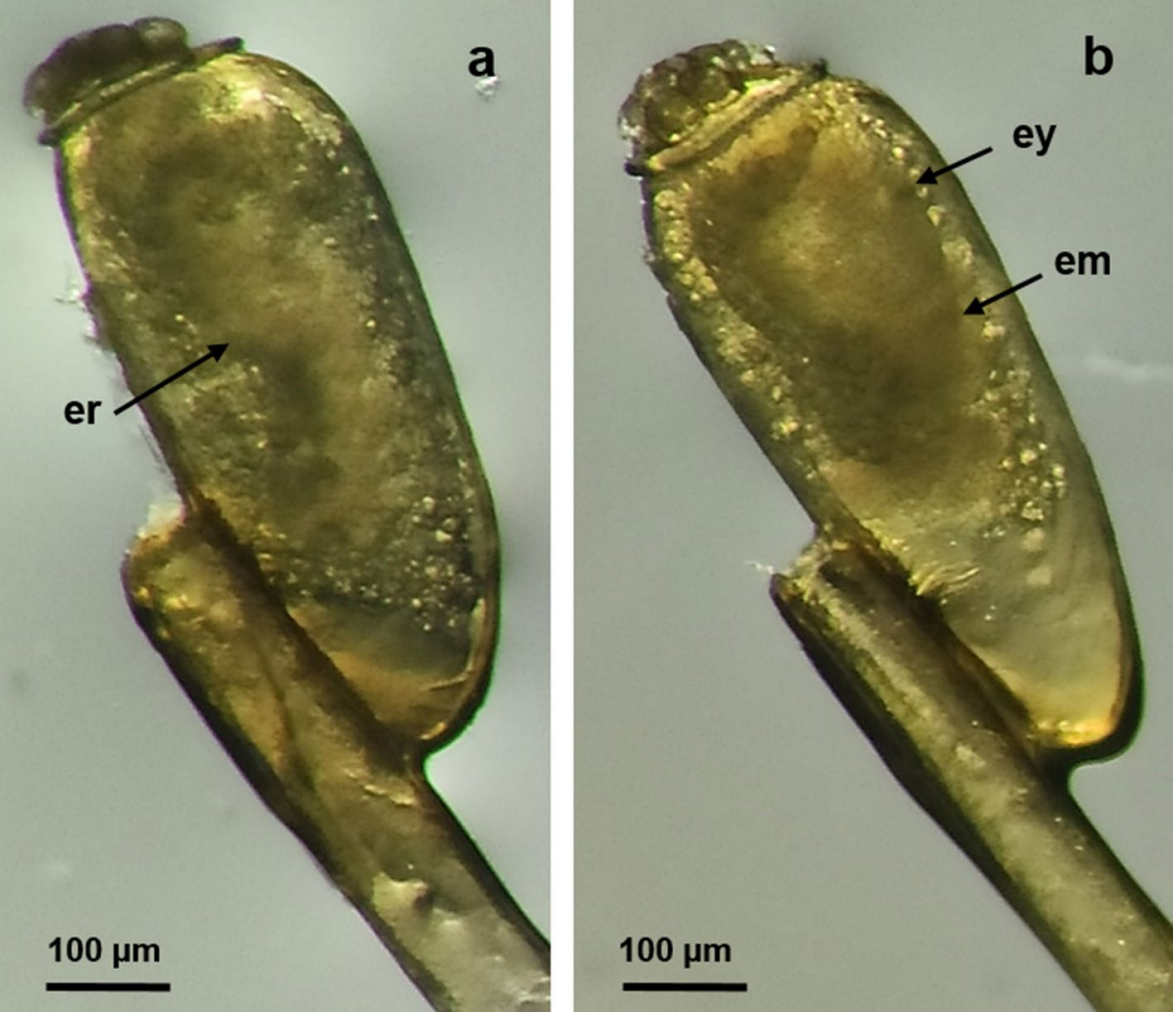
Morphology of medium *P. h. capitis* eggs. **a** Egg with an embryonic rudiment (er). A large opaque area surrounded by yolk granules can be observed; 100× magnification. **b** Egg with a partially differentiated embryo (em). The eye outlines (ey) and less visible yolk granules can be observed; 100× magnification

At this stage, the eggs presented an embryonic rudiment formed from the germ band. The embryonic rudiment was found in the ventral part of the eggs and appeared as a large opaque area surrounded by yolk granules (Fig. 3a). Subsequently, a partially differentiated embryo was formed, presenting two distinct areas: in the larger and more opaque anterior area, two small anterodorsal points representing the eye outlines were observed, while the posterior area of the embryo was darker. The yolk granules started to fade as they concentrated in the embryo while this was defined (Fig. 3b).

#### Late Eggs

**Fig. 4:**
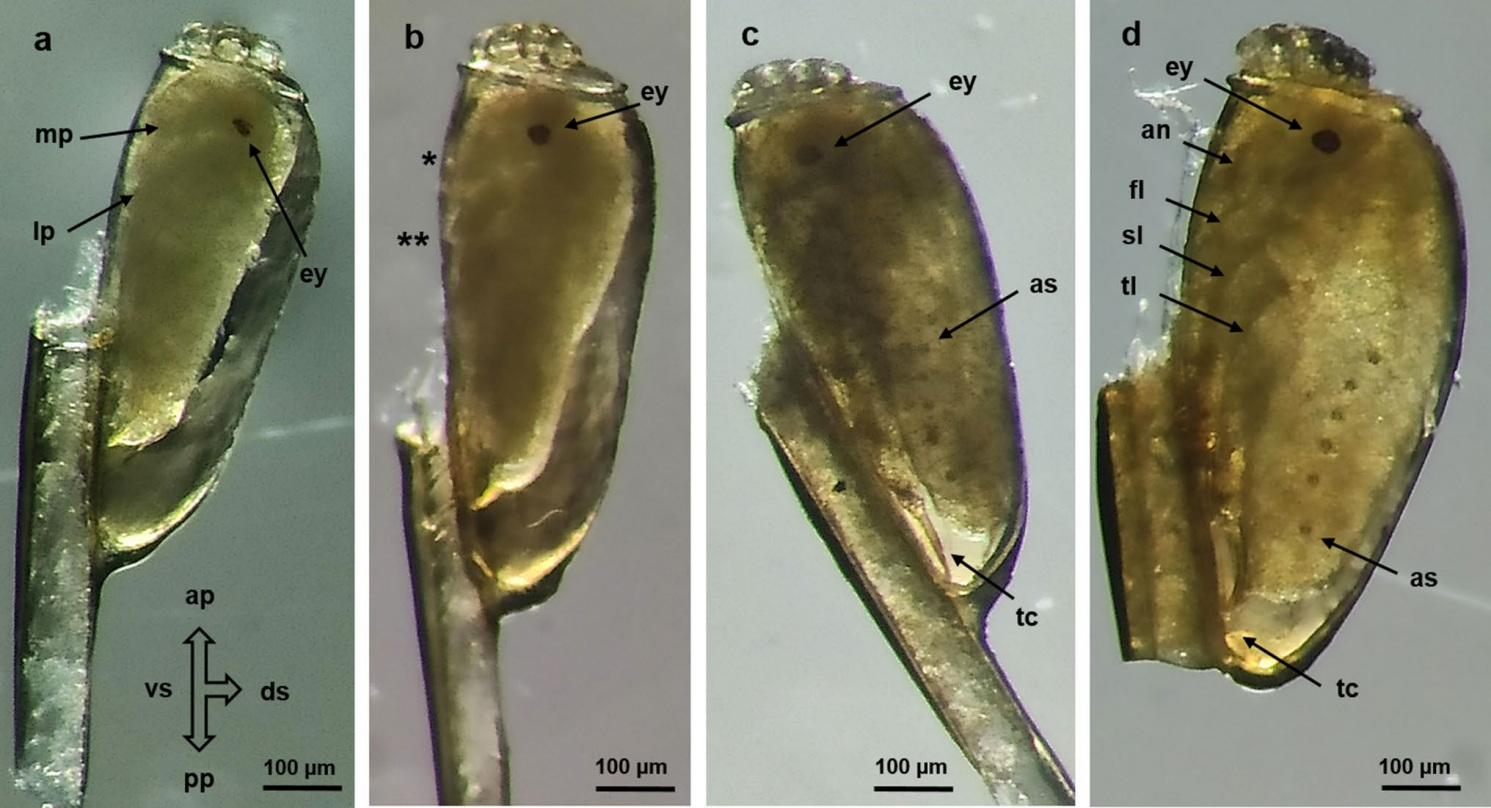
Morphology of late *P. h. capitis* eggs. **a–d** Eggs with visible embryos. **a** The cephalic area, mouth primordium (mp), red eyes (ey), and leg primordium (lp) can be seen; 100× magnification. **b** The mouthparts (*) and legs in development (**) are visible, while the yolk granules surrounding the embryo can no longer be observed; 100× magnification. **c–d** Two large, pigmented eyes (ey), the antennae (an), the first-to-third pairs of folded legs (fl, sl, tl), and the tarsal claw (tc) can be observed; 100× magnification. **d** In the final developmental stage (nymph I stage), all structures and segments are defined: the cuticle is dark and six pairs of marked abdominal spiracles (as) are visible. The nymph I is ready to hatch

In the eggs of this stage, increased organogenesis was observed, with the embryos presenting differentiation and growth. The yolk granules were no longer visible around the embryo as this began to grow and become morphologically defined. In the cephalic area, the mouth primordium was observed, and the eyes appeared as two lateral red dots (Fig. 4a). Gradually, the developing structures, like the mouth cavity and the limbs, were visible (Fig. 4b). The embryo occupied approximately 70% of the egg. The orientation of the embryo followed the dorsal–ventral axis of the egg (Fig. 4a–b).

All segments continued differentiating as the embryo increased in size. The three segments corresponding to the head, thorax, and abdomen were visible. On the head, two pigmented large eyes and the ventrally folded antennae were distinguished. The thorax presented three pairs of well-developed legs with visible tarsal claws (Fig. 4c). On the abdomen, six lateral points that corresponded to the spiracles were identified. In the final phase of development, the embryo occupied approximately 95% of the egg, with a free space in the apical part. The cuticle was dark, and the insect had acquired the morphology of the nymph I stage with the ability to hatch (Fig. 4d).

All the eggs were observed at different angles. However, observation from the side provided greater and more consistent morphological information, possibly coinciding with the position that the larva adopts during its development.

## Discussion

Using stereoscopic microscopy, the external morphology of *P. h. capitis* eggs was described, and many fine morphological characteristics of the embryo were identified through the chorion. The studied sample included empty hatched eggs and non-hatched eggs with a content ranging from fine yolk granules to a developed embryo or, at a low percentage, defective eggs without content. These results highlight the need for large magnification to evaluate the eggs’ development and viability.

In the analyzed sample, 23.26% of the eggs had hatched. As the hair grows, empty eggshells accompany it moving away from the scalp, where nits, a term proposed to refer to the empty eggshell [31], become easier to detect. Although these shells are harmless, they are cosmetically inappropriate according to the modern rules of esthetics. They also indicate prior lice infestation, which can result in stigmatization.

In this study, both viable and defective/non-viable eggs presented an operculum with ten aeropyles, confirming previous reports [30, 31]. In almost all insects, spermatozoa enter the eggs through micropyles [16], with some eggs presenting multiple micropyles [33]. However, in this study, no such structure was identified, following the lack of evidence that exists in the *P. h. capitis*-related literature regarding this structure. This highlights the need to employ other microscopic techniques to study the possible existence, number, and position of micropyles in the egg.

The finding of defective/non-viable eggs could be related to the age of the females. It has been reported that many of the eggs of *P. h. capitis* females that are older than 2 weeks are not viable [8]. In *Musca domestica*, when an ovary has mature eggs, an oostatic hormone is produced to prevent the release of developmental neurosecretory hormone from the egg necessary for vitellogenesis [16]. This suggests that the older females of *P. h. capitis* could activate chemical signals that affect protein synthesis by the fatty body, impeding vitellogenesis in some eggs. Consequently, factors such as the age of female *P. h. capitis* and the possible activation of chemical signals could partially explain the presence of defective/non-viable eggs. Moreover, the low percentage of defective/non-viable eggs in our study could be due to the fact that the sample was oviposited by females of different ages. It should be noted that this is the first study to document these eggs. Future research should consider this finding and conduct novel investigations to clarify the true percentage of defective/non-viable eggs oviposited by females of different ages. In this context, the need for further investigation to elucidate the physiological mechanisms underlying this phenomenon in *P. h. capitis* is crucial.

Embryogenesis has been studied mostly in holometabolous insects, including the fruit fly, *Drosophila melanogaster*, the beetle of the flour, *Tribolium castaneum*, and the moth silk, *Bombyx mori* [34]. The morphological characteristics of *P. h. capitis*’ embryonic development described here permit the general comparison with the cricket’s (*Gryllus bimaculatus*) development [30]. However, several physiological events in head louse development may be specific to their hematophagous nature.

Here, new markers are proposed for the differentiation between the three embryogenesis stages. As for the early eggs, some virgin females of *P. h. capitis* have been considered to lay eggs without fertilization [12], similar to females that have mated [24]. Consequently, we added viability and fecundity markers to this stage. The viability markers involved assessing the external integrity of the egg and its content, i.e., yolk granules, which are the main reserve of nutrients during embryonic development. The fecundity marker is the development of the germ band, which indicates that the egg was fertilized before oviposition. These markers can be observed between days 1–2 post-oviposition [24]. Previously, it was believed that this stage is devoid of markers [25]. However, in this study, we consider that this stage is crucial for the progression of embryogenesis, provided there is an optimal microenvironment for it (Table 1).

**Table 1:** Markers of embryonic development proposed in previous works and the present study

Egg classification	Previously proposed embryonic development markers	Proposed embryonic development markers and markers of viability and fecundity in this study
Early eggs	Fine yolk granule content Formation of yolk masses of variable size [24] Absence of external markers [25]	Abundant fine yolk granules Multiple yolk granules of variable size and a forming germ band* Yolk granules in the center of the egg and an evident germ band* Viability marker: external integrity of the egg and yolk granule content* Fecundity marker: development of germ band*
Medium eggs	Milky, slightly opaque areas Limb formation [24] Reddish eyes and appendages outlines [25]	Embryonic rudiment: large opaque area surrounded by yolk granules* Partially differentiated embryo. Anterior area: opaque and larger, with two small anterodorsal spots (outlines of the eyes); posterior area: darker
Late eggs	The body and the limbs are more clearly defined [24] The eyes are visible and appear as two pink anterolateral spots [24] Black eyes and visible appendages [25]	Defined embryo The yolk granules are not visible around the embryo* ${\text{The embryo occupies approximately 7}}0\% {\text{ of the egg}}^{{\text{*}}} \left\{ {{\text{Cephalic area}},{\text{ red eyes}},{\text{ mouth primordium}},{\text{ leg primordium}},{\text{ mouthparts}},{\text{ and developing legs}}} \right.$ $${\text{The embryo occupies approximately 95}}\% {\text{ of the egg}}^{{\text{*}}} \left\{ {\begin{array}{*{20}c} {{\text{Head}}:{\text{ large pigmented eyes}},{\text{ ventrally folded antennae}}} \\ {{\text{Thorax}}:{\text{ three pairs of well}} - {\text{developed legs}},{\text{ tarsal claws}}} \\ {{\text{Abdomen}}:{\text{ six pairs of spiracles}},{\text{ dark cuticle}}} \\ \end{array} } \right.$$

Data taken from Nuttall (1917) [24] and Cueto et al. (2006) [25]

* Markers proposed

The finding of defective/non-viable eggs allowed the establishment of morphological differences with early eggs that present fine yolk granules. Theoretically, embryogenesis will not be carried out in defective/non-viable eggs due to the apparent absence of yolk, contrary to early eggs. Usually, all eggs that are located at < 1 cm from the scalp are considered alive [27]. This should be taken with caution, and the possibility of finding defective/non-viable and infertile eggs should be contemplated when testing ovicidal drugs.

As for the strengths of this study, it provides a novel description of the embryonic development stages of *P. h. capitis* while confirming the morphological characteristics of medium and late eggs presented in previous reports [22, 23]. Furthermore, we propose additional biomarkers (e.g., the presence of the mouth primordium, spiracles and antennae, the development of tarsal claws, and the proportion of the egg occupied by the embryo) to facilitate the differentiation between stages. These morphological characteristics appear between days 3–4 (medium egg) and 5–7 (late egg) post-oviposition [24]. In this context, it should be noted that the development and hatching rate of the eggs is influenced by the environmental conditions (e.g., temperature and relative humidity), with registered variations dependent on the conducted experiments [22, 24, 26, 34]. Due to the lack of a standardized* in vitro* system for the breeding of head lice, morphological markers of embryonic development are a better reference regardless of the age of the eggs.

Like any study, this work is not devoid of weaknesses, such as the possible bias of information concerning the origin and transport of the samples. As for the origin, we could not collect more data on the environmental and individual variables of the parasitized participants due to the low number of cases. Regarding the transport, after reviewing all transport phases, we found no justification for the presence of these defective/non-viable eggs.

Research on ovicidal activity has made it necessary to update the morphological characteristics of embryogenesis in *P. h. capitis*. Considering that susceptibility to insecticides may vary depending on the embryonic developmental stage [26], this update could be useful and facilitate the standardized evaluation of new drugs with ovicidal activity. In this vein, ovicides could be classified as broad-spectrum, when interrupting the embryogenesis process at all stages, or selective spectrum, when they intervene in specific developmental stages. The majority of studies evaluating drugs for insect control have focused on the adult or immature stage, overlooking the egg stage, resulting in an increase in insecticide resistance. Indeed, this has been observed during the recent emergence of bed bug infestation, *Cimex lectularius* L, and the presence of populations resistant to pyrethroid insecticides [36]. Such resistance has been also documented in insects like triatomine bugs [37]. In agriculture plagues, resistance to common insecticides has increased during the last eight decades [38, 39]. Although some oral medications for human use can affect fecundity and mortality of hematophagous insects, such as *Cimex lectularius L*, the required dosage in most studies reaches the maximum permitted concentration in plasma [40]. Therefore, our findings highlight the relevance of studying embryogenesis as a potential target for ovicidal drugs. This focus could also be applied to other insects of sanitary or agricultural interest, as a strategy for managing and controlling these populations.

## Conclusion

This paper presents an update on the morphology of *P. h. capitis* eggs and the embryonic development characteristics that are visible through the chorion using stereoscopic microscopy. The classification of embryonic developmental stages could be used as a reference for toxicological tests to facilitate the standardized evaluation of the ovicidal activity of new drugs. Our results highlight the need to delve into the description of eggs at a structural and physiological level for a better understanding of embryogenesis. Since the morphological information in the scientific literature is still limited, future studies that elaborate on relevant information concerning embryonic development are necessary for related toxicological tests.

## Data Availability

The datasets used and/or analyzed during the current study are available from the corresponding author on reasonable request.

## References

[1] Galassi FG, Fronza G, Toloza AC, Picollo MI, González-Audino P (2018). Response of *Pediculus humanus capitis* (Phthiraptera: Pediculidae) to volatiles of whole and individual components of the human scalp. J Med Entomol.

[2] Amanzougaghene N, Fenollar F, Raoult D, Mediannikov O (2020). Where are we with human lice? A review of the current state of knowledge. Front Cell Infect Microbiol.

[3] Núñez H, Arriaza B, Standen V, Aravena N (2017). Comparative study of the claws of *Pediculus humanus capitis* between archaeological and modern specimens. Micron.

[4] Pollack RJ, Kiszewski AE, Spielman A (2000). Overdiagnosis and consequent mismanagement of head louse infestations in North America. Pediatr Infect Dis J.

[5] Ibarra J (1988). How to detect head lice: the changing emphasis in health education. Heal Sch.

[6] Mumcuoglu KY, Friger M, Ioffe-Uspensky I, Ben-Ishai F, Miller J (2001). Louse comb versus direct visual examination for the diagnosis of head louse infestations. Pediatr Dermatol.

[7] Balcioglu C, Burgess IF, Limoncu ME, Şahin MT, Ozbel Y, Bilaç C (2008). Plastic detection comb better than visual screening for diagnosis of head louse infestation. Epidemiol Infect.

[8] Lehane M, Lehane M (2005). The blood-sucking insect groups. The biology blood-sucking insects.

[9] Laguna MF, Risau-Gusman S (2011). Of lice and math: using models to understand and control populations of head lice. PLoS ONE.

[10] Burkhart CN, Burkhart CG, Gunning WT, Arbogast J (1999). Scanning electron microscopy of human head louse (Anoplura: Pediculidae) egg and its clinical ramifications. J Med Entomol.

[11] Dutra JMF, Alves AD, Pessanha T, Rachid R, De Souza W, Linardi PM (2014). Prehistorical *Pediculus humanus capitis* infestation: quantitative data and low vacuum scanning microscopy. Rev Inst Med Trop Sao Paulo.

[12] Kim JH, Lee DE, Park S, Clark JM, Lee SH (2021). Characterization of nit sheath protein functions and transglutaminase-mediated cross-linking in the human head louse, *Pediculus humanus capitis*. Parasit Vectors.

[13] Burkhart CN, Burkhart CG (2005). Head lice: Scientific assessment of the nit sheath with clinical ramifications and therapeutic options. J Am Acad Dermatol.

[14] Burkhart CN, Burkhart CG (2007). Fomite transmission in head lice. J Am Acad Dermatol.

[15] Oliveira DMP, Gomes FM, Carvalho DB, Ramos I, Carneiro AB, Silva-Neto MAC (2013). Yolk hydrolases in the eggs of *Anticarsia gemmatalis hubner* (Lepidoptera: Noctuidae): a role for inorganic polyphosphate towards yolk mobilization. J Insect Physiol.

[16] Gillott C, Gillott C (1995). Reproduction. Entomology.

[17] Belles X, Belles X (2020). The hemimetabolan development. Insect metamorph.

[18] Gilbert SF (1997). Developmental biology.

[19] Tahmisian TN (1943). Enzymes in ontogenesis: choline-esterase in developing *Melanoplus differentialis* eggs. J Exp Zool.

[20] Smith EH, Wagenknecht AC (1959). The ovicidal action of organophosphate insecticides. Can J Biochem Physiol.

[21] Smith EH, Salkeld EH (1966). The use and action of ovicides. Annu Rev Entomol.

[22] Smallman BN, Mansingh A (1969). The cholinergic system in insect development. Annu Rev Entomol.

[23] de Villar MIP, Zerba EN, Wood E, de Licastro S (1980). Neurogenesis and occurrence of cholinesterase in eggs of *Triatoma infestans*. Comp Biochem Physiol Part C, Comp.

[24] Nuttall GHF (1917). The biology of *Pediculus humanus*. Parasitology.

[25] Cueto GM, Zerba E, Picollo MI (2006). Embryonic development of human lice: rearing conditions and susceptibility to spinosad. Mem Inst Oswaldo Cruz.

[26] Sonnberg S, Oliveira FA, Araujo de Melo IL, de Melo Soares MM, Becher H, Heukelbach J (2014). *Ex vivo* development of eggs from head lice (*Pediculus humanus capitis*). Open Dermatol J.

[27] Barker SC, Altman PM (2011). An *ex vivo*, assessor blind, randomised, parallel group, comparative efficacy trial of the ovicidal activity of three pediculicides after a single application - melaleuca oil and lavender oil, eucalyptus oil and lemon tea tree oil, and a “suffocation” pediculicide. BMC Dermatol.

[28] Al-marjan K, Koyee Q, Abdullah SMA (2015). *In vitro* study on the morphological development of eggs (nits) and other stages of head lice *Pediculus humanus capitis* De Geer, 1767. Zanco J Pure Appl Sci.

[29] Bowles VM, Yoon KS, Barker SC, Tran C, Rhodes C, Clark MJ (2017). Ovicidal efficacy of Abametapir against eggs of human head and body lice (Anoplura: Pediculidae). J Med Entomol.

[30] Donoughe S, Extavour CG (2016). Embryonic development of the cricket *Gryllus bimaculatus*. Dev Biol.

[31] Maunder JW (1983). The appreciation of lice. Proc R Inst G B.

[32] Mehlhorn H, Abdel-Ghaffar F, Al-Rasheid KAS, Schmidt J, Semmler M (2011). Ovicidal effects of a neem seed extract preparation on eggs of body and head lice. Parasitol Res.

[33] Resh V, Cardé R (2009). Encyclopedia of insects.

[34] Panfilio KA (2008). Extraembryonic development in insects and the acrobatics of blastokinesis. Dev Biol.

[35] Devore CD, Schutze GE (2015). Head lice. Pediatrics.

[36] Gonzalez-Morales MA, Romero A (2019). Effect of synergists on Deltamethrin resistance in the common bed bug (Hemiptera: Cimicidae). J Econ Entomol.

[37] Fabro J, Sterkel M, Capriotti N, Mougabure-Cueto G, Germano M, Rivera-Pomar R (2012). Identification of a point mutation associated with pyrethroid resistance in the para-type sodium channel of *Triatoma infestans*, a vector of Chagas’ disease. Infect Genet Evol.

[38] Pérez CJ, Alvarado P, Narváez C, Miranda F, Hernández L, Vanegas H (2000). Assessment of insecticide resistance in five insect pests attacking field and vegetable crops in Nicaragua. J Econ Entomol.

[39] Metcalf RL (1989). Insect resistance to insecticides. Pestic Sci.

[40] Sheele JM, Ridge GE, Du W, Mallipeddi N, Vallabhaneni M (2017). A screen of pharmaceutical drugs for their ability to cause short-term morbidity and mortality in the common bed bug, *Cimex lectularius* L. Parasitol Res.

